# Persistent Disability Six Months after Initial Disability Less Likely in Older Women

**DOI:** 10.1093/geroni/igab046.3185

**Published:** 2021-12-17

**Authors:** Raj Shah, Katherine Webb, Joanne Ryan, Rory Wolfe, Michael Ernst, Sara Espinoza, Robyn Woods

**Affiliations:** 1 Rush, Rush University Medical Center, Illinois, United States; 2 Monash University, Melbourne, Victoria, Australia; 3 University of Iowa, Iowa City, Iowa, United States; 4 University of Texas Health Science Center San Antonio, San Antonio, Texas, United States

## Abstract

Many community-dwelling older adults develop activity of daily living (ADL) disability and subsequently regain function. Using data from the ASPirin in Reducing Events in the Elderly (ASPREE) clinical trial, we examined the relationship of gender, incident disability, and persistent disability 6 months after the incident disability. Walking, bathing, dressing, transferring, toileting, and eating were assessed as ADLs, at bi-annual interviews. ADL disability was defined as requiring help with or inability to do or severe difficulty with ≥1 ADL; persistent disability was an ADL loss at 6 months after a first (incident) ADL disability. Discrete time, multivariable Cox proportional hazards regression was utilized to estimate associations with developing incident ADL disability described as cause-specific hazard ratios, with death as a competing outcome. For persons with incident ADL disability, odds of developing persistent disability at 6 months as compared to recovery was determined using multivariable logistic regression. These analyses included 18,414 (51.6% women) ASPREE participants in the United States and Australia aged 70+ years (65+ years if U.S. ethnic minority) without ADL disability at trial entry. During a median follow-up of 4.7 years, 1,485 participants (63.2% women) developed an incident ADL disability, and, of those, 272 (57.0% women) met criteria for persistent disability at 6 months. Women had an increased risk (HR=1.17, 95% CI=1.05 to 1.32) of developing incident ADL disability; however, women were less likely to have persistent disability versus recovery 6 months later (OR=0.66, 95% CI=0.49 to 0.89). Why persistent disability development is lower in older women needs further exploration.

